# Combined Low-Level Light Therapy and Intense Pulsed Light Therapy for the Treatment of Dry Eye in Patients with Sjögren's Syndrome

**DOI:** 10.1155/2021/2023246

**Published:** 2021-06-10

**Authors:** Matteo Di Marino, Paola Conigliaro, Francesco Aiello, Claudia Valeri, Clarissa Giannini, Raffaele Mancino, Stella Modica, Carlo Nucci, Roberto Perricone, Massimo Cesareo

**Affiliations:** ^1^Ophthalmology Unit, Department of Experimental Medicine, University of Rome Tor Vergata, Rome 00133, Italy; ^2^Rheumatology, Allergology and Clinical Immunology, Department of Medicina Dei Sistemi, University of Rome Tor Vergata, Rome 00133, Italy

## Abstract

**Purpose:**

To evaluate the effects of combined intense pulsed light therapy (IPL) and low-level light therapy (LLLT) in dry eye disease (DED) in patients affected by Sjögren's syndrome. *Patients and Methods*. This is a monocentric, prospective, interventional study. At baseline, all the study patients (*n* = 20) were on tear substitute therapy and underwent Schirmer type-1 test and breakup time (BUT) test. After baseline measurements, tear substitute therapy was suspended, and patients underwent IPL and LLLT. The same investigations were carried out at one (T1) and at three (T3) months after treatment. The Ocular Surface Disease Index (OSDI) survey was used to measure the severity of DED.

**Results:**

BUT test showed an increase in tear film breakup time in patients with DED 1 month after the beginning of the treatment (T0 vs T1: *p*=0,01). This increase was even more statistically significant after 3 months of the IPL and LLLT treatment (T0 vs T3: *p* < 0.0001). Schirmer test values increased too, but there was not statistically significance between values at T0 and T1 or T3. The patients perceived an improvement in their condition, which resulted in a lower score on the OSDI survey. The OSDI score was lower at T1 than T0 (T0 vs T1: *p*=0.0003), while it tended to increase again after 3 months although it was still lower than baseline (T0 vs T3: *p*=0.02). No facial or ocular side effects were reported.

**Conclusions:**

The use of combined IPL/LLLT for the treatment of DED in patients affected by Sjögren's syndrome appears to be beneficial.

## 1. Introduction

Dry eye disease (DED) is a multifactorial disorder involving different components of the tear film and the ocular surface. It provokes discomfort and visual acuity alterations and, in the absence of adequate treatment, can lead to ocular surface damage [1]. The lacrimal film has three main components: an aqueous component produced by both principal and accessory (Krause and Wolfring) lacrimal glands, a mucinic component produced by the goblet cells, and a lipid component produced by the Meibomian glands located in the eyelids. According to some authors, the aqueous and mucin layers should be considered as a single, “inseparable” layer of mucoaqueous gel [1].

In most cases, DED is due to functional alterations of the Meibomian glands. When this occurs, the lipid component of the tears decreases, resulting in excessive lacrimal evaporation. In fact, it has been observed that an insufficient or absent lipid layer can increase lacrimal evaporation, thereby affecting lacrimal osmolarity and promoting eye inflammation [2, 3].

Sjögren's syndrome (SS) is a systemic chronic autoimmune inflammatory disease [4]. It is characterized by lymphocyte infiltration into exocrine glands, especially lacrimal and salivary glands.

SS can occur as a primary disease or be associated with other autoimmune diseases [5].

The factors most strongly associated with impaired health-related quality of life (HRQoL) in patients with SS were patient-reported symptoms, especially the dry eye severity [6, 7].

Up to date, the first line of treatment for DED in SS has been the topical application of lacrimal substitutes [8]. Various studies and Cochrane reviews support the daily use of topical lacrimal substitutes for the symptomatic relief of dryness, with a significant improvement in HRQoL without significant side effects. There are several commercially available products, with different compositions, costs, and ease of administration, which should also be considered when suggesting a therapeutic strategy. Second line approaches include autologous serum eye drops, corticosteroid, and cyclosporin A topical therapies, while the occlusion of lacrimal puncta, resulting in increased tears permanence in the eye, and oral administration of pilocarpine, that increases the secretion of the aqueous components, are indicated as rescue therapies [8, 9]. Ocular occlusion or protection with contact lenses should be considered only when other therapies failed, or in case of imminent risk of corneal damage [10].

In the last years, new medical devices have been developed with the aim of reducing the evaporation of the tear film in the pathogenesis of the DED. These instruments use specific wavelengths to selectively stimulate the Meibomian glands and to activate their metabolism [11–15].

Intense pulsed light (IPL) is a technology based on a polychromatic light (wavelength spectrum between 500–1200 nm, modulated through a filter) that induces a selective photothermolysis of the irradiated tissue. The thermal impulses stimulate the Meibomian glands to restart their normal activity. Applied on the periorbital region and cheekbones, the light stimulates the contraction of the glands, thereby increasing the lipid flow and its liquefaction. The lipid compartment stabilizes the aqueous component with consequent reduction of the lacrimal evaporation. This photobiomodulating technique has been used for many years in several fields of medicine (i.e., dermatology) [16–18].

Potential mechanisms whereby IPL could achieve therapeutic efficacy include improvement of rosacea disease by thrombosis of abnormal blood vessels below the skin surrounding the eyes, heating the Meibomian glands and liquefying the meibum, activation of fibroblasts and enhancing the synthesis of new collagen fibers, eradication of Demodex and decreasing the bacterial load on the eyelids, interference with the inflammatory cycle by regulation of anti-inflammatory agents and matrix metalloproteinases (MMPs), reducing the turnover of skin epithelial cells and decreasing the risk of physical obstruction of the Meibomian glands, and changes in the levels of reactive oxygen species (ROS) [19]. The low-level light therapy (LLLT) is a particular type of photobiomodulation, based on light-emitting diodes. An athermal and atraumatic cellular photoactivation leads to a significant improvement in tear breakup time (BUT). Moreover, LLLT therapy has proven effective in patients affected by Meibomian gland dysfunction [20].

The aim of this study is to evaluate the effects of this combined therapy in DED in patients affected by SS.

## 2. Materials and Methods

This is a monocentric, retrospective study approved by the Institutional Review Board, with all researches adhering to the principles outlined in the Declaration of Helsinki (registration number in the register of the Independent Ethics Committee of the PTV Policlinico Tor Vergata Hospital R.S.216.17). Written consent was obtained for each participant. Patients with SS were recruited in consecutive and perspective manner from the Rheumatology Clinic of Policlinico Tor Vergata Hospital and referred to the Ophthalmology Clinic of the same hospital. Diagnosis of SS was made according to 2016 EULAR/ACR classification criteria, including BUT <10 s, Schirmer I (no anesthesia) <5 mm of the paper after 5 min, and ocular surface assessment by staining (Oxford scale grade ≥2, Van Bijsterveld score ≥4 in both eyes). [21].

Inclusion criteria: (1) diagnosis of SS; (2) age >18 and <75 years old; and (3) use of artificial tears and lubricant at baseline. Exclusion criteria: (1) contact lens wear; (2) any ocular surgery within the last 6 months; (3) acute or chronic ocular disease other than dry eye disease; (4) previous diagnosis of allergic conjunctivitis; (5) concomitant autoimmune disease except SS; (6) use of any other topical treatment, except for lacrimal substitutes; and (7) documented history of photosensitivity.

None of the patients suffered from manifesting Meibomian gland dysfunction. All patients suspended the therapy with lacrimal substitutes before starting treatment. The Eye-light® (Espansione Marketing SpA, Bologna, Italy) is a new device that allows performing both IPL and LLLT therapies [15].

**Figure 1:**
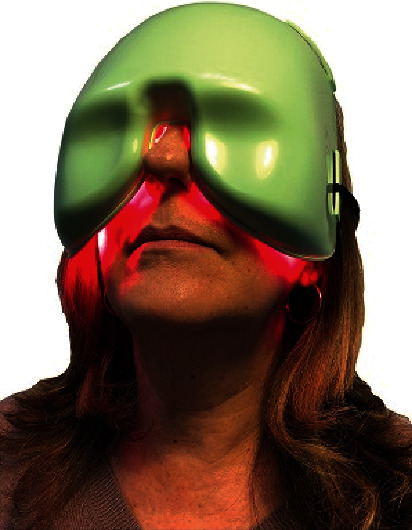
LLLT treatment.

20 SS patients underwent 4 cycles of combined treatment. Subjects were automatically classified by the instrument in one of the phototype available categories, from the lightest to the darkest phototype, according to the Fitzpatrick score, in order to adapt the intensity of the following treatment [15]. An automated software adjusts the optimum therapeutic energy level (10–16 joules/cm^2^). Unlike other IPL instruments, the use of gel is not required with the Eye-light system. A patented cooling system using forced air maintains the temperature of the crystal at a nondamaging level calibrated on the patient's skin type. Each treatment was carried out in a supine position, and the patient's eyes were covered with a protective device during the application of IPL. Five applications were performed on each eye to the lower periorbital area using the 12 cm^2^ delivery system. Three applications were performed along the inferior orbital rim, ensuring placement to the inferior edge of the lid margin while protecting the globe. A fourth application was delivered vertically near the lateral canthus while the last one was performed with the device horizontal along the inferior orbital rim. This sequence takes about 5 minutes for both eyes. Then, the LLLT treatment was performed applying a special mask for 15 minutes (Figure 1) (wavelength of 633 ± 10 nanometers; emission power of 100 mW per cm^2^; total fluence in the treated area: 110 Joules per cm^2^). No protective eyewear is indicated during this procedure. However, the patients were instructed to keep their eyes closed to maximize the LLLT effect on the upper and lower lids. Every session is designed to both stimulate the function of the Meibomian glands and to soften the meibum. Sessions have been performed weekly for one month.

### 2.1. Ophthalmological Evaluation

All study patients underwent the following tests at baseline while on tear substitute therapy and after one and three months of IPL and LLLT treatment but off therapy with lacrimal substitutes. Patients were advised to discontinue tear substitute therapy after baseline measurements.

Schirmer type-1 test: a 30–35 mm × 5 mm bibulous paper strip was introduced in the lower conjunctival fornix of the temporal side of both eyes in the absence of anesthetic drops. The test lasted 5 minutes. Normal values range from 10 to 30 mm. Values equal or lower than 5 mm are considered pathological and indicative of lacrimal hyposecretion [22].

BUT test has been performed on both patients' eyes. The test was repeated 2-3 times per eye, after a single fluorescein strip application, to obtain an average BUT time for each eye. A BUT time greater than 10 seconds was considered normal, while a BUT time lower than 5 seconds was deemed to be clinically altered [23, 24].

Ocular Surface Disease Index (OSDI) questionnaire was used for the screening and the diagnosis of ocular surface alterations. It allows quickly evaluating the severity of the symptoms and the impact that the dry eye has on the daily life of the patient. All patients were asked 12 questions concerning 3 different DED parameters: symptomatology, visual function, and environmental influences. A score ranging from 0 to 100 is assigned to each patient. The higher the score, the higher the severity of the dry eye. The score severity was then subdivided into 4 grades: absent (0–12), mild (13–22), moderate (23–32), and severe (33–100) ocular surface disease [25, 26].

All measures were collected at the same time of the day by the same expert ophthalmologist (MC).

### 2.2. Statistical Analysis

Descriptive statistics are presented as means − standard deviations. Outcome measures before and after treatment were analyzed using the Wilcoxon signed-rank two-tailed test (nonparametric). Statistical significance was set at the *p*=0.05 level. All calculations were carried out using GraphPad software (Prism version 8.0, California, USA).

## 3. Results

### 3.1. Population Characteristics Are Resumed in Table 1

**Figure 2:**
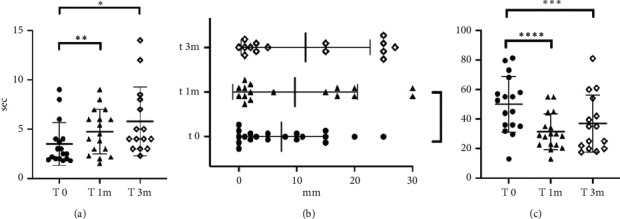
Representative values at different time points. (a) BUT values. (b) Schirmer test values. (c) OSDI values.

BUT test showed an increase in tear film breakup time in patients with DED evident at 1 month (T1) after the beginning of the treatment (T0 vs T1: *p*=0.01; Figure 2(a)). This decrease was even more statistically significant after 3 months of the treatment (T0 vs T3: *p* < 0.0001; Figure 2(a)).

Schirmer test values increased too, but there was no statistically significant difference between values at T0 and T1 or T3 (Table 2).

Patients perceived an amelioration in their condition upon the combined treatment, which resulted in a lower score at the OSDI survey (Figure 2(c), Table 2). The OSDI score associated with the symptoms was decreased immediately after the end of the treatment (T0 vs T1: *p*=0,0003; Figure 2(c)), while it tended to increase again after 3 months although it was lower compared to baseline (T0 vs T3: *p*=0.02; Figure 2(c)). There were no reported facial or ocular side effects.

Based on the OSDI evaluation, at the baseline (T0), 2 patients revealed a moderate DED (10%), while 18 (90%) showed a severe disease.

At T1, 2 patients showed mild DED (10%), 8 a moderate DED (40%), and 10 (50%) a severe disease.

At T3, 6 subjects (30%) reported mild DED, 2 a moderate form (10%), and 12 (60%) a severe disease.

## 4. Discussion

The dual stimulation of the Meibomian glands with both IPL technology and LLLT aims at reducing the evaporative component of DED [15]. We tested their combined efficacy on a group of SS patients and observed both objectively measurable and patient-perceived improvements. BUT test showed an increase in the lacrimal film stability while OSDI survey revealed a decrease of the subjective discomfort complained by the patients. Although the improvement was more evident at the end of the treatment (at 1 month), it was still observable at the 3-month follow-up time point. It is important to notice that all study subjects except one have completed the experimental therapeutic protocol without the necessity to reintegrate the lacrimal substitutes. Schirmer test remained stable overtime; this result could be explained by the fact that the treatment does not stimulate the Krause and Wolfring glands and the main lacrimal glands, and only the evaporative component of dry eye is involved. Despite this, the different DED subtypes are not mutually exclusive [15]. Although SS historically affects the exocrine glands, and not the sebaceous ones (such as the Meibomian glands) [27], several studies showed the contemporary presence of a Meibomian gland dysfunction in patients with SS [28, 29].

It is not clear if the presence of lymphocyte infiltration in the sebaceous glands is confirmed [30], but the Meibomian glands of patients with SS are impaired more severely than the glands of dry eye patients without SS, and this impairment seems to be more severe when the diagnosis of SS has been present for more than 3 years [31, 32]. In our patients, the lacrimal composition was clearly improved after IPL and LLLT therapy, probably due to the increase of the lipidic component. However, it is not possible to exclude some kind of effect also on the other lacrimal glands. The suspension of the therapy with lacrimal substitutes was therefore probably compensated by the effect of the combined treatment, although it did not achieve BUT, Schirmer, and OSDI values of nonpathological subjects.

In our study, the Eye-light instrumentation has been proven safe and effective. DED patients often perceive the multiple daily administration of artificial tears as tedious, time-consuming, and cost-ineffective, and this may decrease both the quality of life and the compliance to the therapy [11]. With a reduction in the number of therapeutic sessions and of the associated costs, the IPL therapy could thus represent a viable therapeutic option in the treatment of DED patients. The use of this new technology could be effective in the cotreatment of complex pathologies such as SS, in which the amelioration of the evaporative component of the dry eye could improve patient's quality of life.

This study encompasses several limitations as the small number of enrolled patients, the short time of evaluation, and the subjective nature of OSDI. However, precisely because of the OSDI subjectivity, this test can be useful in understanding how the quality of life of these patients and their comfort changed after the treatment. Moreover, due to the small number of participants in this pilot study, no correlations were made between clinical and laboratory characteristics with treatment effects.

## 5. Conclusions

Although the number of patients and the follow-up time are limited, these preliminary results suggest a clear benefit that can be obtained in treating the evaporative component of the dry eye with the IPL and LLLT stimulation of the Meibomian glands in these patients. Randomized controlled trials are necessary to better elucidate the role of combined techniques to optimize patient management.

## Figures and Tables

**Table 1:** Characteristics of the study population.

Female (n/%)	18 (90%)
Age (years)	57.7 ± 19.3
Disease duration (months)	142.1 ± 100.4
ESSDAI	1.4 ± 1.9
Anti-Ro positivity (n/%)	4 (25%)
Anti-La positivity (n/%)	3 (15%)
Positive biopsy (n/%)	6 (30%)
HCQ (n/%)	5 (25%)
ESR	33.3 ± 23
CRP (mg/dL)	0.3 ± 0.4
DMARDs (n/%)	9 (45%)

Note: data are expressed with mean and standard deviation unless differently specified. HCQ, hydroxychloroquine; DMARDs, biologic disease-modifying antirheumatic drugs; ESR, erythrocyte sedimentation rate; CRP, C-reactive protein test.

**Table 2:** Variables under consideration at different time points.

Measure	T0	T1	T3
BUT (sec)	3.5 ± 1.65	4.5 ± 2	5.3 ± 2.7
Schirmer (mm)	8.6 ± 7.9	9.5 ± 10	10.38 ± 9.97
OSDI	50.5 ± 17.5	31.46 ± 12.11	38.31 ± 19.35

Note: data are expressed with mean and standard deviation unless differently specified. BUT, tear breakup time; OSDI, Ocular Surface Disease Index.

## Data Availability

The data used to support the findings of this study are included within the article. Additional data are available from the corresponding author upon request.
